# 3D-printed nanomedicines for cancer therapy

**DOI:** 10.2144/fsoa-2024-0039

**Published:** 2024-05-15

**Authors:** Surbhi Bisht, Sweta Kanwal, Balasubramanian Mythili Gnanamangai, Swati Singh, Devayatbhai Baku Mansi, Ranvijay Kumar, Mayank Sharma, Piyush Kumar Gupta

**Affiliations:** 1 Department of Life Sciences, Sharda School of Basic Sciences & Research, Sharda University, Greater Noida, 201310, Uttar Pradesh, India; 2 Department of Biotechnology, K.S. Rangasamy College of Technology, Tiruchengode, Namakkal, 637215, Tamil Nadu, India; 3 Centre for Nanotechnology, Indian Institute of Technology Roorkee, Roorkee, 247667, Uttarakhand, India; 4 Department of Mechanical Engineering and University Centre for Research & Development, Chandigarh University, Mohali, 140413, Punjab, India; 5 Department of Pharmaceutics, SVKM's NMIMS School of Pharmacy & Technology Management, Mukesh Patel Technology Park, Shirpur, 425405, Maharashtra, India; 6 Centre for Development of Biomaterials, Sharda School of Basic Sciences and Research, Sharda University, Greater Noida, 201310, Uttar Pradesh, India; 7 Department of Biotechnology, Graphic Era (Deemed to be University), Dehradun, 248002, Uttarakhand, India

**Keywords:** 3D printing, cancer, nanomedicine, therapy

Among the malignant diseases, cancer is one of the main causes of the rising death rates in the vast majority of countries [1]. Surgery, radiation therapy, chemotherapy and immunotherapy are just a few of the cancer therapeutic modalities that have evolved considerably over the years. According to estimated projections in 2020, there will be 19.3 million new instances of cancer worldwide with 10 million deaths associated with it [2]. Each year, approximately 400,000 children develop cancer, and it is expected that by 2040, there will be 29.5 million new cancer cases, resulting in 16.4 million fatalities.

In the past 10 years, significant progress has been made in the treatment of cancer. Healthcare professionals acknowledge and commonly employ traditional cancer therapy as the benchmark for addressing cancer treatment. These are often referred to as conventional approaches in the realm of cancer therapy, especially amid the introduction of innovative techniques in the contemporary era [3]. Even though surgery, chemotherapy and radiation remain the mainstay of traditional cancer treatment, continuous efforts to identify the most efficient approaches for addressing the disease are an ongoing drive. A major barrier to the therapeutic application of efficacious anticancer drugs is a disparity between *in vitro* and *in vivo* evaluations. Therefore, current drug development methods must be substituted with physiologically appropriate *in vitro* models that replicate the cancer milieu. To address this challenge, 3D-bioprinting has surfaced as a promising technique. This procedure makes use of biomaterials, which include cells and other physiologically helpful substances necessary for cell growth and development.

In 3D bioprinting, biological structures are constructed by employing biomaterials which include cells and other biologically effective components that are required to promote cell growth and development, replicating the intricate details of live tissue. This technology can be utilized to print implants and biocompatible surgical guides using a diverse range of materials. As a result, the effectiveness of existing products will improve. Thus, 3D printing is a valuable technique for accelerating the initial phases of product development in less time and cost. The first US-FDA-approved 3D-printed dosage form was SPRITAM, a levetiracetam pill that degrades quickly [4].

## 3D-printed nanomedicines for cancer treatment

3D printing is an approach for producing 3D objects through computer-aided design (CAD) [5]. It has contributed by giving relevant data for research and the production processes of several areas, such as medicine, pharmaceuticals, aviation, ecological monitoring and automobiles. 3D printing was invented by a scientist, Charles Hull in 1986 using a process called stereolithography (SLA). Techniques for printing in three dimensions are incredibly inventive and have progressed to a versatile scientific state. To enhance cost and efficiency in industrial manufacturing, 3D printing connects novel opportunities and concepts. The components utilized in 3D printing applications include metal, graphene-based materials, thermoplastic polymers and other materials [6]. Nowadays, in 3D printing technology, researchers can look into an array of medical-related alternatives, including the rapidly growing areas of drug delivery methods, tissue design, organ and tissue models, prostheses and replica fabrication, inserts and more, as they look to exploit 3D printing to produce oral dose forms with different shapes and sizes. Nanomedicine, involving the integration of nanotechnology in multiple medical domains like medication repurposing, theranostic instruments, and site-specific drug delivery, prompts researchers to explore innovative approaches like multimodal targeting, stimuli-responsive release and theranostic applications. This exploration involves combining nanomaterials to create hybrid materials [7,8]. Additionally, nanomedicines hold enormous potential for a variety of theranostic applications in infectious diseases. They may avert the transmission of infectious diseases, brain tumors, multidrug-resistant tumors, metastatic tumors and relapsed tumors that pose major obstacles for clinical studies. Owing to their extraordinarily small size and narrow size distribution, various forms of nanomaterials, including nanoparticles (lipid, polymer and metallic), nanodiamonds, nanocrystals, dendrimers, quantum dots (QDs), carbon nanotubes (CNTs), nano gels, nanoemulsions and many more have been explored for the development of various forms of nanomedicines. Furthermore, these nanomedicines are being developed as precision medications with 3D printing technology to fulfill the distinctive requirements of every individual at a personalized level. This is achieved by combining bioconjugation, exceptional biocompatibility and apparent surface functional design which made 3D printing technology an efficient cancer therapy tool for various cancer treatments.

For instance, Wang *et al.*, fabricated a 3D printing technique that enabled osteosarcoma to be treated with customized local chemotherapy. Several *in vivo* experiments were carried out that closely resembled actual clinical chemotherapy conditions. They demonstrated that 3D printed poly L-lactic acid (PLLA) implants may be engineered with precisely regulated physical morphologies and capable of programming microprobe structures, resulting in excellent carriers for anticancer medications [9].

In a study Chen *et al.*, evaluated the function of Cu/AG hydrogel scaffolds with chemo-photo thermal effects that were constructed via 3D printing technology. Under NIR irradiation, the Cu^2+^-loaded hydrogel scaffolds showed outstanding photothermal effect and *in vitro* release kinetics. This suggests that the integration of copper ions and NIR treatment was effective in eradicating thyroid cancer cells and cancer organoids isolated from patient samples. Notably, the result showed that the combined use of Cu^2+^-based PTT and CDT enhances therapy efficacy while overcoming the drawbacks of using a single cancer treatment strategy [10].

Zheng *et al.*, employed 3D printing technology to synthesize a breast mold. Using this printed breast mold, they crafted a polydimethylsiloxane (PDMS) prosthesis that incorporated microspheres containing paclitaxel (PTX) and doxorubicin (DOX). Biochemical characteristics of the generated prosthesis including its shape, drug loading effectiveness, encapsulation effectiveness and *in vitro* drug absorption, were assessed. The prosthesis's efficacy for therapy and systemic toxicity was examined using a mouse model of breast cancer. The findings of the study showed that the microspheres of PTX and DOX placed into the 3D-printed prosthesis could deliver the medications continuously for almost 3 weeks, inhibiting cancer recurrence with minimal adverse effects [11].

Moreover, by employing extrusion-based 3D printing poly (lactic-co-glycolic acid) (PLGA) films synthesized with either lidocaine alone or in combination with paclitaxel and rapamycin. Every layer contains either low or high molecular weight PLGA. The drug release kinetics of paclitaxel, rapamycin and lidocaine for PLGA films were analyzed *in vitro* and contrasted with PLGA hydrogel discs composed of polyethylene glycol (PEG) and PLA. The findings indicate that the drug-polymer interactions used molecular models and simulations to explain drug release attributes examined *in vitro*. Additionally, drug release behaviors from polymeric films and discs have also been identified via drug solubility in water, and drug position within the polymeric matrix, based on model analysis [12].

Yang *et al.*, formulated an efficient drug delivery system that reduces concentration while boosting drug effectiveness in treating malignant breast cancer. E-jet 3D printing was employed to generate controlled-release polylactic acid/glycolic acid (PLGA) scaffolds to administer doxorubicin (DOX) and cisplatin (CDDP) sequentially. Compared with the results of individual drugs and the results when two medications were delivered without PLGA scaffolds, this drug delivery method permitted the use of a lower drug dosage, which led to a greater effect on the death of human breast cancer cells and inhibition of tumor growth. Based on the studies, DOX-CDDP-PLGA scaffolds efficiently eliminate MDA-MB-231 cells and prevent the formation of tumors [13].

Landgraf *et al.*, validated a 3D-printed drug delivery system (3D-PDDs) to enhance the local therapeutic efficacy of frequently administered chemotherapy drugs in bone tumors to minimize their systemic adverse effects. The investigation of the 3D-PDDs indicated that the implant could effectively halt cancer infiltration, leading to localized tumor cell death near the scaffolds without causing adverse systemic effects. The study evaluated the impact of locally applied 3D-printed doxorubicin (DOX)-loaded medical-grade polycaprolactone (mPCL) scaffolds in a humanized primary bone cancer model. These findings collectively demonstrate the potential of 3D-PDDs as a therapeutic and diagnostic tool in an orthotropic humanized osteosarcoma (OS) tumor model [14].

The application of additive manufacturing techniques, such as 3D printing, has enabled the fabrication of 3D items that have contributed to personalized medicine [15]. Based on its affordability, ease of use and versatile application, 3D printing has garnered significant momentum in the drug-delivery system, especially in cancer nanomedicine. As the field and methods of vascular imaging and drug-delivery systems are evolving, the use of nanomedicine is also actively contributing to the development.

## Conclusion & future perspective

With ongoing evolution of lifestyles, people are progressively emphasizing the maintenance of advanced medications over maintaining a balanced diet. This trend has significantly contributed to the exponential growth of 3D-printed formulations. Recently, researchers have utilized 3D printing to provide numerous dosages while minimizing material wastage. This approach helps in improving pharmacokinetics, facilitates design of innovative drug formulations and support combination therapies. These strides have significantly improved the medicine and healthcare system and developed personalized drugs and medical transportation systems. Considering these advancements, 3D printing has become a valuable tool in the progress of personalized medicine for cancer treatment and tailoring medications to meet individual patient's specific needs. Moreover, this technology is also required to develop three-dimensional culture models for testing newly developed anti-cancer drugs.
